# Supplementary figures and images for: Characterization of oral swab samples for diagnosis of pulmonary tuberculosis
Source: PLoS One. 2021 May 17;16(5):e0251422. doi: 10.1371/journal.pone.0251422 (PMC8128230; doi:10.1371/journal.pone.0251422)

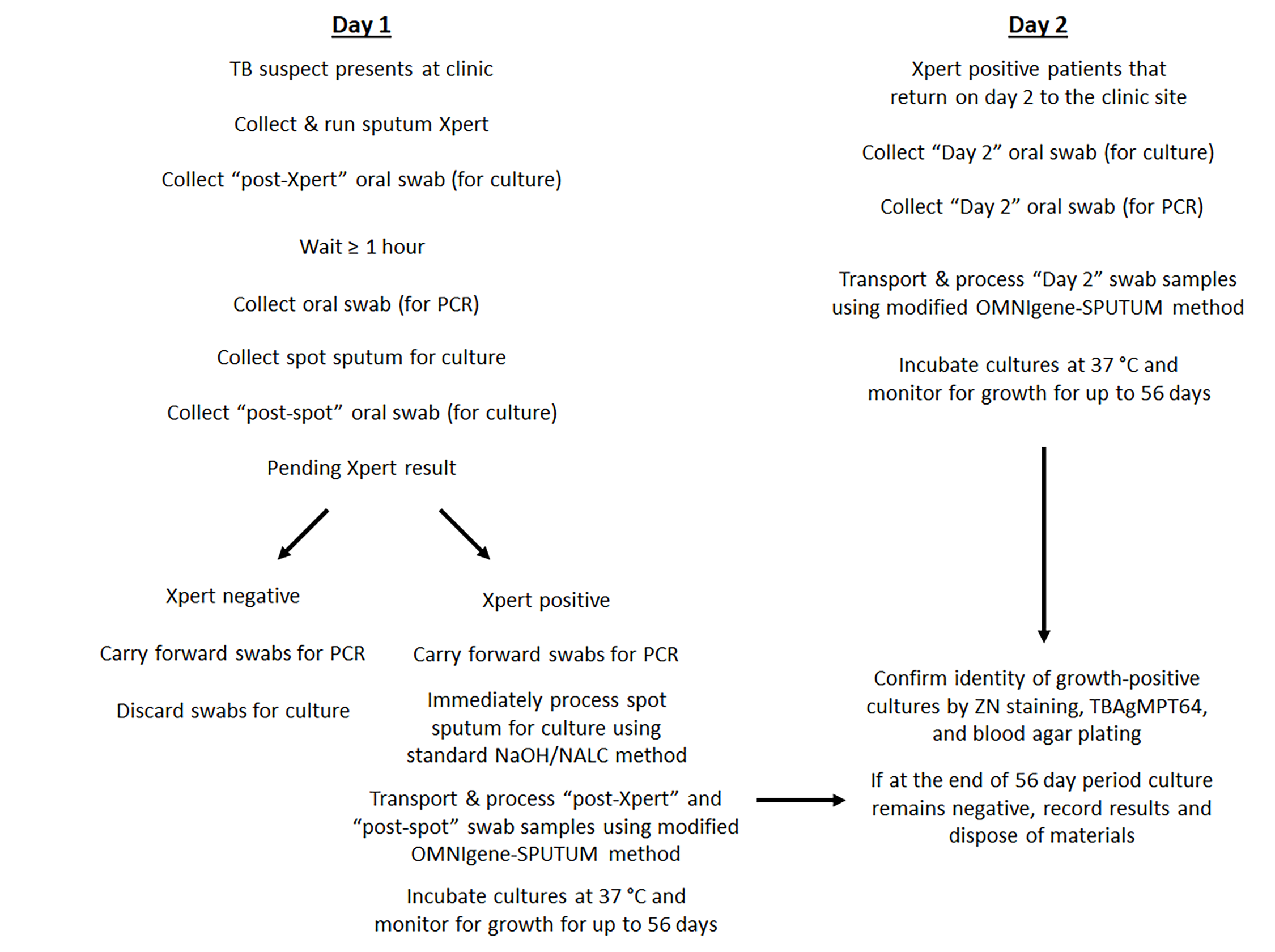

Supplement: S1 Fig — (TIF) [file pone.0251422.s002.tif]
