# Supplementary material for: Characterization of oral swab samples for diagnosis of pulmonary tuberculosis
Source: PLoS One. 2021 May 17;16(5):e0251422. doi: 10.1371/journal.pone.0251422 (PMC8128230; doi:10.1371/journal.pone.0251422)
Supplement: Attachment — Submitted filename: Response to Reviewers.docx [file pone.0251422.s003.docx]

PONE-D-20-28429 Manuscript Re-submission

**Point-by-point response to reviewer comments**:

Reviewer #1:

Abstract: “Sensitivities of the collection methods are not sufficient for use in routine culture based diagnostic tesating” is one of the main findings, but the abstract gives a different view, and conveys the message that this collection procedure will be an alternative to the rest stating that it might facilitate culture based diagnostic testing for TB. This needs to be rephrased in a manner so that the reader might get a true picture.

The positive findings of the study related to the comparison of the results with molecular based techniques are all reported previously and the abstract reiterates the same. The only positive finding is that viable bacilli is present in tongue, but this is expected and the authors have tried to merely substantiate this. But this message is not seen in the abstract.

Please review revised abstract in which we have focused more on the qPCR/molecular results that are now central to this manuscript. The information regarding culture positivity is included as an arm of the study, but without reference to using oral swab cultures for diagnosis.

Materials and methods: (Line 110 ) It is mentioned that all the cultures were incubated for 56 days, But is it the same for MGIT systems also?
MGIT cultures were incubated for 42 days. We have revised the text to make this clear (Line 171).

Results and Discussion: (Line 133) It is not clear if the figure 61.4% includes single oral swab culture results or both, do specify this.

The original text as written indicated “While the collection design for Days 1 and 2 differed with respect to preceding sputum production, 87 of 141 (61.7 %) patients had MTB-positive tongue swab cultures when considering all samples together.” We have revised this language to clarify: “While the collection design for Days 1 and 2 differed with respect to preceding sputum production, 87 of 141 (61.7 %) patients had MTB-positive tongue swab cultures when considering all samples together.” (Lines 335 – 337).

Table 3 legend shows swab culture, but is not reflected in the table title
In the revised manuscript these data are included as Table 6, and we have revised the table title to clarify based on this point: “**Table 6. Correlation between swab culture and smear microscopy grade or GeneXpert semiquantitative result.**”

(Line 163) This statement is contraindicative, says it is not statistically significant in the second half, but the sentence begins that the assessment is most discriminatory and how do you justify that the second day specimen is able to do so and the reason behind this.

The first half of the sentence was intended a comment that the Day 2 samples (collected without prior prompted sputum production) are more likely to be reflective of a patient’s natural disease state, while the observation that there was not a significant difference between Day 2 (no prior prompted sputum) and Day 1 samples (prior prompted sputum) is a comment on study design. We have modified the language in the revised draft, which can be found on lines 396-400.

Line 169 is confusing and explain what does oral sampling rate stands for.

Thank you for raising this. This was a word omission on our part, and should have read “…oral sampling culture contamination rate…”. With the focus on the qPCR/molecular results in the revised manuscript we have removed this text.

Reviewer #2: This is an interesting but a simplistic and short study. In this reviewer's opinion it does not reach the level of a research article - a short communication would be more appropriate. The following are to be noted:

1. There is no clear description of the clinical application of the oral swab method.

We have addressed this concern in the revised manuscript with a greater focus on the diagnostic potential of qPCR from oral swab sampling. The integration of this information can be found across the manuscript; however, we specifically point out Lines 63 – 81 in the Introduction, the sociodemographic and clinical characteristics of the patients included in the study (Tables 1 & 2), comparison to GeneXpert of swab-based molecular MTB diagnosis (Tables 3 & 4), the text throughout the Results section associated with these tables, and the expanded text in the Discussion (for example, Lines 362 – 367 and Lines 406 – 413).

2. No clear description/implications or insights for the understanding of TB pathogenesis presented/discussed.

In the revised manuscript we have shifted the emphasis from insights on TB pathogenesis attendant with the phenomenon of culturable MTB on the tongue dorsum, to a molecular diagnostic focus. We believe the culture/viability data still support the observation that knowledge of viable MTB on the tongue contributes to our knowledge base of the pathogen, but as the reviewer points out in the absence of additional data we limit further speculation on TB pathogenesis.

3. The authors are respectfully advised to substantially expand the scope of the paper for it to fly as a full fledged research article.

Thank you for this comment and suggestion. In line with other reviewers and editor suggestion, we hope that our revised manuscript and expanded scope satisfy this request.

4. On p3, lines 51-54 the tongue swab procedure is described stating three swabs were taken. The first and the third swabs are mentioned clearly but when was the 2nd one taken? It is not clear.

We have added a supplemental figure, “Fig S1” with a complete workflow of sample collection.

5. Table 1 shows Day2 culture positivity for the one swab sample taken is 43.9%. The authors have not discussed why they think it is so much lower than the first swab on Day 1 (50.4).

The information raised in this comment can now be found in Table 5 and is described in the text on Lines 335 – 336 and Lines 393 – 401, in which we suggest that while it there is not a statistically significant difference in culture tongue swab culture positivity with or without preceding sputum production, it is possible that this procedural detail in the sample collection workflow (see Figure S1) explains the discrepancy.

Reviewer #3: The manuscript “Viable Mycobacterium tuberculosis from swabs of the tongue dorsum of
pulmonary tuberculosis patients” is quite interesting and informative.

The paper is simple and concise but few more additions seems imperative before it can be considered for publication. The study emphasizes on the use of non-sputum method in the context of use in paucibacillary situation as an alternate to sputum. There are few clarifications needed.

1. Apart from culturing of MTB, was there any molecular diagnostic test performed on these tongue swabs? The gene Xpert results used for selection criteria is performed on the sputum samples and not the tongue swabs. If this method is going to be suggested as an alternate to sputum collection a parallel rapid diagnostic test to first identify positive and then its co-relation to their growth as culture will be more valid.

Consistent with comments from other reviewers, we have substantially revised the manuscript to include molecular/qPCR results to address this concern.

2. The bacilli were cultured in MGIT, LJ and 7H10. It would be very useful to give a comprehensive table in place of table 2 given currently to understand the revival and contamination rate in different MTB media assessed here.

With the revised focus on molecular detection of MTB from tongue swab, we felt that the content of the former table 2 distracted from the new focus. We have removed this table from the manuscript.

3. It would be interesting to add the drug resistance data on the isolates and any indications to cultivability.

We agree that these are interesting questions; however, in our study setting, focused drug resistance (as determined by Xpert Rif analysis) is quite low (2 of 141 individuals, 1.4 %), and with the sample size it is difficult to draw significant conclusions on this question. This may be an excellent subject for follow-up studies, perhaps with a larger sample size and/or in settings with a greater burden of drug resistant MTB.

Reviewer #4: The authors in this manuscript describe an approach for isolation of viable Mycobacterium tuberculosis from swabs of the tongue dorsum of pulmonary tuberculosis patients. The study looks interesting, but the authors should address the following concerns.

• The results indicate that viable mtb bacilli do exist in the tongue dorsum, but the culture positivity is quite low. How will it improve the current TB diagnosis portfolio? Where do the authors want to position this test? Molecular tests are gaining advantage and use of DNA isolated from these samples as an input in endorsed molecular tests will provide a platform for positioning this technique.
We appreciate this comment and agree with the reviewer’s assessment that molecular tests are gaining advantage in MTB diagnosis. Our revised manuscript is updated accordingly. We have retained data regarding viability of MTB collected with tongue swabs (Tables 5 & 6, with associated text Lines 319 – 346), however our focus is on the operational rather than diagnostic implications of these data (see, for example, Lines 401 – 405).

• It will be useful to see the contamination rates of OminiGene vs. the NALC-NaOH method for the sputum versus swabs, as the former is reported to provide lower contamination rates, but here the rates were quite high (130/477 were contaminated). It will be useful to standardize the decontamination procedure for tongue swabs.

While we retain our data on the viability of MTB from tongue swabs, our revised manuscript focuses on the ongoing efforts to develop (and ultimately standardize) oral swab analysis for molecular detection of MTB. We agree that if any tongue swab method were to be adopted for routine use, standardization of methodsis a critical component.

• Was the sample stored after reconstitution in OMNIgene tube? Did that make a difference in culture positivity? What advantage does the author foresee for this method? It will be interesting to see the viability assessment of bacilli stored in the OMNIgene tube over a period of time.

We have clarified the description of the methods to address this point (see Materials & Methods, Lines 183 - 196). The swab samples were stored in OMNIgene-SPUTUM for 18-24 hours at 25 °C prior to sample processing and culture inoculation.

• Line 192. What bio-safety measures do the authors suggest, in view of their results. Also, a follow-up experiment might be useful to see how does the mtb load decrease in the dorsum during the course of therapy.

While a data-driven answer to the question of MTB load over the course of therapy was outside the design and scope of this analysis, we agree that this is an interesting question for follow up work, as it may offer insights into the efficacy of a given therapy particularly in cases of drug resistance. While we were unable to conduct those experiments with our patient cohort we hypothesize that oral swab culture positivity rates over the course of treatment would track with sputum culture positivity rates.

• What was interesting was the number of samples positive by swab culture (42.4%) in smear-ve category; what category of Xpert result did these samples lie in? A head to head comparison of Xpert versus Smear grade in all samples might be useful.

We agree that these data reinforce the observation that MTB culture is more sensitive than smear microscopy, and can now point to tongue swab as an additional sampling matrix that follows this pattern; however, we feel that a head-to-head comparison of sputum Xpert results versus sputum smear microscopy falls outside of the scope of the current manuscript. We note, here, that for the majority of individuals (8 of 14) from this smear negative group had “very low” or “low” Xpert results, and the remainder (6 of 14) were split evenly between medium and high Xpert results.
