# Supplementary material for: Characterization of oral swab samples for diagnosis of pulmonary tuberculosis
Source: PLoS One. 2021 May 17;16(5):e0251422. doi: 10.1371/journal.pone.0251422 (PMC8128230; doi:10.1371/journal.pone.0251422)
Supplement: Attachment — Submitted filename: Wood et al PONE-D-20-28429R1 Response.docx [file pone.0251422.s004.docx]

PONE-D-20-28429R1 Manuscript Re-submission

**Point-by-point response to reviewer comments**

**NOTE:** Line numbers in our responses refer to the mark-up version. Major changes are also highlighted in the mark-up version.

Reviewer #3:

1. The paper titled “Characterization of oral swab samples for diagnosis of pulmonary tuberculosis” is a neat study. In the present form it lacks being presented as a full-length article only based on methodology and its results. However, with the variety of papers that characterise oral swabs for diagnosis published in last 5 years, the authors need to justify how this study is different and how this study is a value addition to the previous ones. The scope of the paper in comparison to previously published ones is still lacking. The results need to be discussed in detail in context of previous published studies and not just the opinion of the authors about the same in the present context. There is no insight on the recommendations for culture of mycobacteria and preferred method for maximum recovery of viable bacteria among the three.
   - We thank the reviewer for this summary of critiques, and point to several areas in the manuscript where their concerns are addressed. We note that key differences that distinguish this work from previous studies are articulated in the abstract (Lines 38-41, highlighted in the Track Changes version): “In previous analyses, qPCR testing of swab samples collected from tongue dorsa was up to 93 % sensitive relative to sputum GeneXpert, when 2 swabs per patient were tested. The present study modified sample collection methods to increase sample biomass and characterized the viability of bacilli present in tongue swabs.” We note further that we discuss this study/these results in comparison to previous work in several other locations in the manuscript (see, for example, lines 88-95 and in several highlighted passages in the Discussion).
   - The reviewer also flags that there is no insight on the recommendation for culture of mycobacteria. As discussed in the cover letter associated with the manuscript resubmission, following the reviews of our original submission, we substantially revised the manuscript to focus on the molecular detection of MTB from oral swabs, rather than broad diagnostic application of culture-based methods. With that revised focus, in the present manuscript we do make note of considerations for occupational safety, and suggest that the demonstration that at least some DNA collected from the tongue is associated with whole, viable MTB cells has implications for sample handling and processing (Lines 407-411).
2. Line 49 Make it uniform % (87/114) to make it easy to understand.
   - The text has been updated.
3. Line 36 refers to geneXpert while line 45, 47 and 288 refers to GeneXpert Ultra, please give correct detail. Is it MTB/RIF or Ultra? Throughout the manuscript it reads MTB/Rif – eg Line 120 as the first portion was processed for Xpert (Xpert MTB/RIF assay, Cepheid, Sunnyvale, CA, USA).The authors got to be specific.
   - The comment on Line 36 refers to results published in a different study, and in that study the authors used sputum GeneXpert (as stated in the abstract). Within the current manuscript, we implemented the nomenclature suggested by the reviewer (GeneXpert MTB/Rif Ultra) throughout the manuscript. This information is also reflected in columns G and H in Table S1. For consistency, we have added the designator “Ultra” to line 126.
4. Line 51 – Referring to oral swabs as a screening in asymptomatic patients? While line 63 says “oral epithelium during 64 active TB disease” Line 71 says “useful for diagnosis and screening in non-clinical and community settings” Please clarify
   - Line 63 is a description of previously published data. In this study our study enrollment criteria were to include patients “…who presented with respiratory symptoms…” (Line 107), and so we do not make comment on the screening of asymptomatic patients in the abstract. To minimize confusion we changed the wording that the reviewer flags to: “useful for TB case finding in non-clinical and community settings” (Line 76).
5. Line 101 adults (>18 years) – please provide interquartile range for this.
   - The requested data are included in Table 1. The text on Lines 96-104 is a description of our prospective study design and inclusion criteria.
6. Line 190-192 Clarify - the incubation periods for LJ and MGIT or 7H10 are not the same.
   - As described in Lines 198-199, and in Fig S1, in this study all cultures from tongue swab were incubated for up to 56 days. We acknowledge that guidance documents indicate different incubation periods for the various media, however, in this study investigating the tongue as a source matrix for viable MTB we adopted a conservative, long, incubation/observation period of 56 days uniform across all media types. As described in the section titled “Sputum sample processing and analysis,” for sputum culture, we incubated (sputum) MGIT cultures for up to 42 days and (sputum) LJ cultures for up to 56 days (lines 177-179).
7. Line 238-240 - Based on Cq values from qPCR 239 analysis, Puritan PurFlock Ultra swabs were found to collect about twice as much MTB DNA as Whatman OmniSwabs® 240 (p = 0.015). Kindly discuss this in comparison to previous reported studies.
   - This sentence refers to results reported previously (reference 4, Luabeya et al 2019), as noted in the preceding sentence. The complete passage reads “Previously, we compared two swab brands for their abilities to detect MTB DNA on buccal (not tongue) surfaces in the mouths of adult TB patients. Based on Cq values from qPCR analysis, Puritan PurFlock Ultra swabs were found to collect about twice as much MTB DNA as Whatman OmniSwabs® (p = 0.015) [4].”
8. Line 365 This was a different population from previous studies, 366 and we did not do side-by-side clinical comparisons between the two swab brands. Please add further discussion to the swab type variation in comparison to previous studies.
   - These comparisons and context are presented throughout the current manuscript. Please see, for example:
     - The paragraph on Lines 77-88
     - Lines 370-372 “…previously it had taken 2 swab per patient… the current study exhibited up to 94 % sensitivity relative to sputum culture using just one swab per patient.”
9. Fig S1 refers to MGIT or LJ or both. Please confirm reference to 56 days wait for confirming culture negative.
   - This comment is addressed in response #6, above.
